# Optical imaging of muons

**DOI:** 10.1038/s41598-020-76652-8

**Published:** 2020-11-26

**Authors:** Seiichi Yamamoto, Kazuhiko Ninomiya, Naritoshi Kawamura, Yoshiyuki Hirano

**Affiliations:** 1 Department of Integrated Health Science, Nagoya University Graduate School of Medicine, Nagoya, Japan; 2 Department of Chemistry, Osaka University Graduate School of Science, Osaka, Japan; 3 Muon Science Laboratory, IMSS, KEK, Tsukuba, Japan

**Keywords:** Experimental nuclear physics, X-rays, Experimental particle physics

## Abstract

Optical imaging of particle beams is a promising method for range and width estimations. However it was not clear that optical imaging was possible for muons. To clarify this, we conducted optical imaging of muons, since high-intensity muons are now available at J-PARC. We irradiated positive muons with different momenta to water or plastic scintillator block, and imaged using a charge-coupled device (CCD) camera during irradiation. The water and plastic scintillator block produced quite different images. The images of water during irradiation of muons produced elliptical shape light distribution at the end of the ranges due to Cherenkov-light from the positrons produced by positive muon decay, while, for the plastic scintillator block, we measured images similar to the dose distributions. We were able to estimate the ranges of muons as well as the measurement of the asymmetry of the direction of the positron emission by the muon decays from the optical images of the water, although the measured ranges were 4 mm to 5 mm larger than the calculated values. The ranges and widths of the beams could also be estimated from the optical images of the plastic scintillator block. We confirmed that optical imaging of muons was possible and is a promising method for the quality assessment, research of muons, and the future muon radiotherapy.

## Introduction

Optical imaging is a promising approach for quality assessment (QA) of high energy X-rays from linear accelerators (LINAC)^1–7^. Optical radiation imaging mainly measures Cherenkov-light from electrons or secondary electrons produced in water. Recently, the luminescence of water at a lower energy than the Cherenkov-light threshold for particle ions was found and has been used for dose and range estimations^8–11^. Luminescence of water was also found in X-ray, alpha particles, and beta particles^12–16^. Although optical imaging for dose and range estimation was tried for these radiations, it has not been tried for muons.

A muon is a particle similar to an electron, with an electric charge of e^−^ for a negative muon and e^+^ for a positive muon; however, its mass is 207 times that of an electron. A muon has a mean life-time of 2.2 μs and a negative muon decays to one electron and two types of neutrinos ($\overline{\upnu }$_e_ and ν_µ_), while the positive muon decays to one positron and two types of neutrinos ($\nu$_e_ and $\overline{\upnu }$_µ_). Since these characteristics are quite different from familiar radiations such as X-rays, electrons, protons, or carbon ions, new results for such applications for QA, research, or radiation therapy may be obtained by the optical imaging of muons. For the high-energy cosmic muons, they are used for the radiography of huge subjects such as a volcano dome^17^, pyramid^18^ or nuclear reactor^19^, which is impossible to image with other methods.

High-intensity muon beams are now available in an experimental facility at the Japan Proton Accelerator Research Complex (J-PARC)^20,21^ and pulsed positive or negative muon beams are now used in applications such as nondestructive X-ray fluorescence spectroscopy^22–27^. Efficient QA of the beams is desired for some of these applications. In addition, effective QA will be important when muons are used for radiation therapy in the future^28,29^. However, there is no such QA method for muon beams. To date, only a trial for the lateral beam profile measurement of muons using a scintillator has been conducted^27^.

We previously used Monte Carlo simulation to calculate the dose and light distributions of positive muons in water to determine the feasibility of experimental optical imaging of muons for dose or range measurements^30^. In this simulation, dose distribution was given mainly by muons, while the light distribution attributed to the positrons produced by muon decay^30^. Since we found that the positive muons of J-PARC have enough intensity for the optical imaging experiments, we conducted the imaging of the muons. Here, we show the first optical images measured during irradiation of muons to water and a plastic scintillator block.

## Materials and methods

We used positive muons with momenta of 73.9-MeV/c, 84.5-MeV/c, and 95.1-MeV/c and the deviation of the momentum was 4%. We used positive muons for our imaging experiments because the intensities of the positive muons at J-PARC were higher than that of negative muons and radionuclide production by irradiation to materials is negligible for positive muons.

1. Experimental set-up for imaging of muon beams.
  Figure 1(A) shows a schematic of optical imaging during irradiation of muons to water. A phantom containing distilled water and a cooled charge-coupled device (CCD) camera (BITRAN, BU-56DUV, Japan) were placed in a black box. The size of the phantom was 10 cm × 10 cm × 10 cm. The phantom was made of 0.5-cm-thick black acrylic board except for the side facing the CCD camera. The side facing to the CCD camera of the phantom was made of a 5-mm-thick transparent acrylic plate (Kuraray PARAGLAS UV00, Japan). Positive muons were irradiated from the side of this phantom as shown in Fig. 1(B) and imaged with the cooled CCD camera from 90 degrees of the muon beam direction. We used a C-mount F-1.4 lens (Computar, Japan) for the CCD camera. The distance from the phantom surface to the lens of the cooled CCD camera was 30 cm. The side of the black box was made of thin black paper to minimize energy loss of the muon beam by the black box. We used the water phantom to image the Cherenkov-light of the positrons produced by the decay of muons^30^.

**Figure 1:**
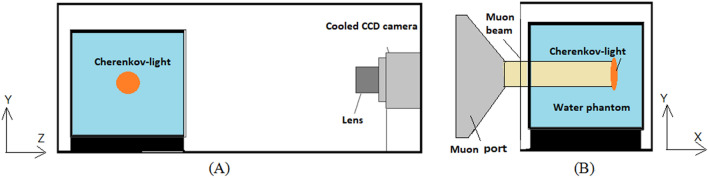
Schematic of optical imaging of water during irradiation of positive muons; side view (**A**) and front view (**B**).
  We also used a 10 cm × 10 cm × 10 cm plastic scintillator block (EJ-200, Eljen Technology, USA) for the muon imaging. Since the plastic scintillator block emits higher light than the Cherenkov-light by the positrons produced by the muons’ decay, we expected to image the dose distributions of the muons using the plastic scintillator block^30^.
2. Imaging experiments.
  In Fig. 2, we show a photo taken during our optical imaging experiments of the positive muons. The black box contained a CCD camera and one of the phantoms, and was installed in the muon beam exit of D1 area at muon facility of J-PARC. Muons were irradiated to the water or plastic scintillator block inside the black box from the left side of the black box, as shown in Fig. 2. The diameter of the muon beam collimator at the beamline exit was 40 mm. The positive muon beam momenta used for the imaging experiments were tuned to be 73.9-MeV/c, 84.5-MeV/c, and 95.1-MeV/c. A positive muon beam was irradiated to either the water or plastic scintillator block and imaging was conducted. The beam intensity of the positive muon beams was ~ 5 × 10^6^ muons/s.

**Figure 2:**
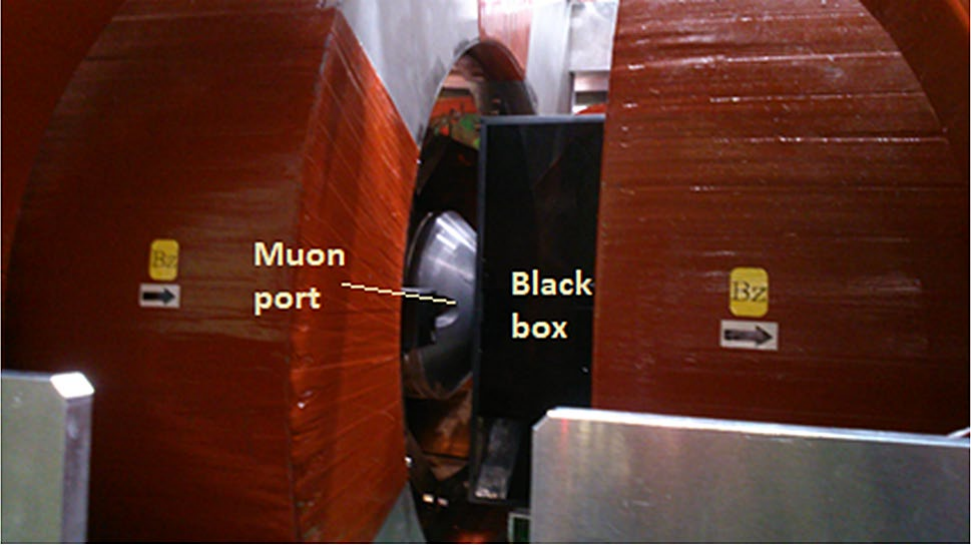
Photo of optical imaging experiments during irradiation of positive muons.
  For the water, we conducted imaging by the CCD camera for 300 s during muon beam irradiation. For the plastic scintillator block, we conducted imaging for 10 s. The calculated dose for the irradiated area in water (300 s) was ~ 0.3 Gy and that for plastic scintillator (10 s) was 0.01 Gy. The image size of the CCD camera was 680 × 512 and the pixel size was 0.34 mm × 0.34 mm.
3. Image evaluation.
  All measured images by the CCD camera during muon irradiation were processed using public domain software (ImageJ). We removed the noise spots using the function of the software (Remove Outlier) produced by direct detections of the CCD image sensor from the scattered muons and escaped positrons, as well as annihilation photons, based on high-intensity and small-pixel information. Then, we subtracted the background image from each obtained image to remove the offset value and non-uniformity of the CCD camera. We also used the software to calculate the depth and lateral profiles. We estimated the absolute size of the images by measuring the known size of the phantom images. We changed all profile data measured on the images to absolute sizes with this value.
  We compared the measured images with those calculated by Monte Carlo simulation. We used Geant4 (version 10.4.2) as the Monte Carlo simulation because it is utilized in many subjects and its quality is evaluated extensively. For electromagnetic processes, we activated “G4EmStandardPhysics_option4”. Processes of ionization, bremsstrahlung, pair production, multiple scattering, Coulomb scattering were included for muon. Geant4 also includes the optical photon processes (G4OpticalPhysics) with Cerenkov-light (G4Cerenkov) and scintillation photon generation (G4Scintillation) in the library. As the light production from the luminescence of water at lower energy than the Cerenkov-light threshold^8–13^, we used the scintillation process. These were the same procedures as we conducted the simulation for positive muons^30^.
4. Possible applications of the measured optical images.
  For the possible applications of the measured optical images of muons in water and plastic scintillators, we conducted range determination of the beams, deviation determination of the momenta of muon beams, and evaluations of the asymmetry of the direction of the positron emission from the muon decays.
  - Beam momentum determination from the images.
    Beam momentum determination is a time and labor consuming task thus efficient method is desired. We made a look up table (LUT) between the muon momentum and the peak depths from the simulated muon images for water. Then we checked the LUT accuracy using the measured muon images for water.
  - Deviation determination of the momentum of muon beam.
    The transported muon beam is not mono energetic but has several percent of momentum bite. Deviation determination of the momentum of muon beam is important but it is also a time and labor consuming task. We made a LUT between the percent deviation of the muon momentum and the widths using the simulated muon images for water. Then we checked the LUT accuracy using the measured muon images for water.
  - Measurement of the asymmetry of the direction of the positron emission from the muon decays.
    It is known that the fraction of the positrons produced by the muon decays emit in forward direction is higher than that of backward^26^. The asymmetry of the directions of the positron emission attributes to the spin polarization of muons. Measurement of the asymmetry of the directions of the positron emission from the muon decays is important in some of the applications and researches of muons^26,31,32^. For this purpose, using the CCD camera, we measured the optical image of water during irradiations of positive muon with the momentum of 84.5-MeV/c for 10 min. The measurements were repeated, and five images were added to form a 50 min acquisition image to reduce the statistical deviation in the image. Then we measured the distances in the profile of the image at half maximum of the peak in forward and backward directions. We compared these distances to measure the asymmetry.

## Results

**Figure 3:**
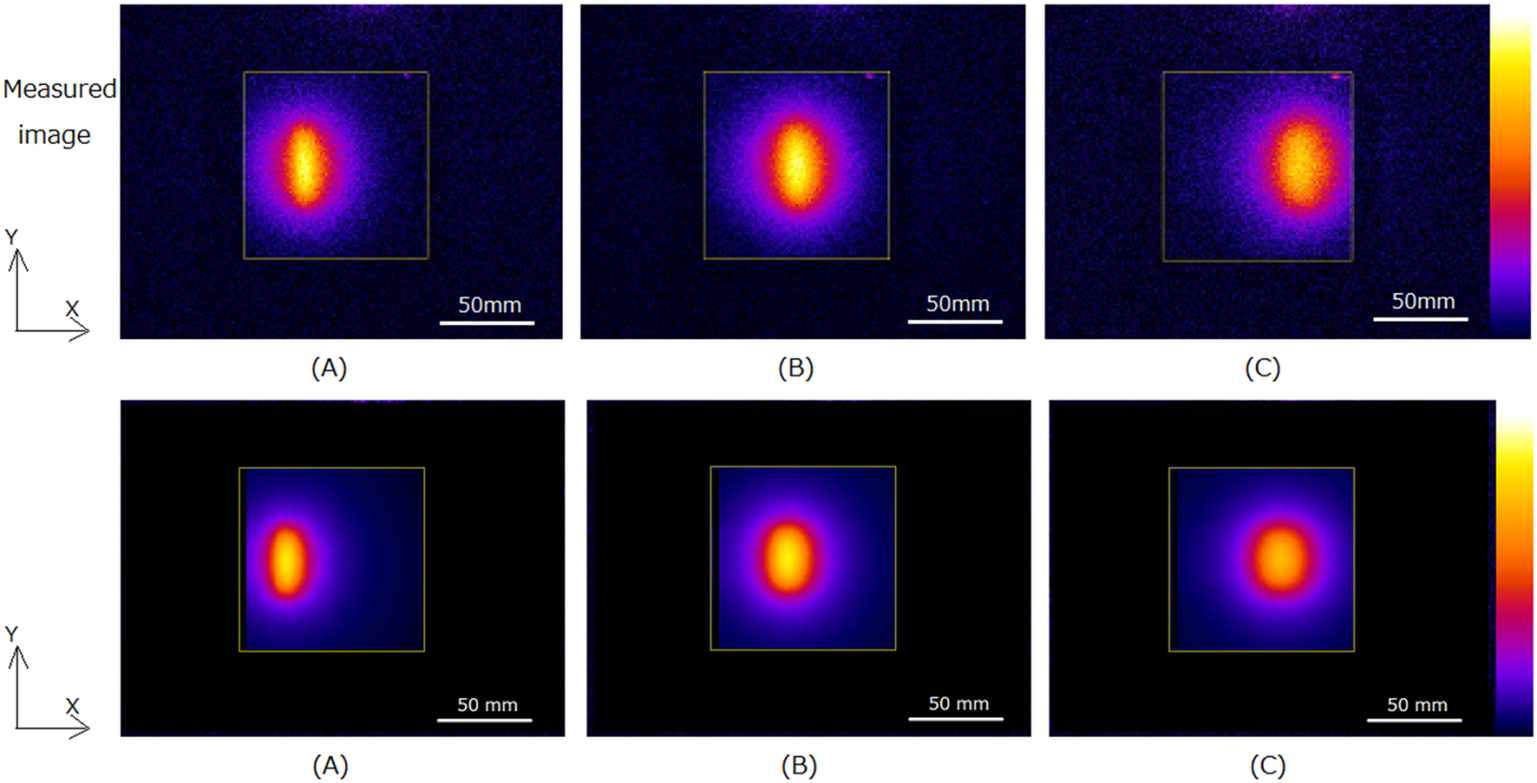
Measured optical images of water (upper part) during 73.9-MeV/c (**A**), 84.5-MeV/c (**B**), and 95.1-MeV/c (**C**) irradiation of positive muons. Yellow squares in each image show the contours of the water phantoms. Beams were irradiated from left side of phantom. Simulated optical images of water (lower part) during 73.9-MeV/c (**A**), 84.5-MeV/c (**B**), and 95.1-MeV/c (**C**) irradiation of positive muons are also shown.
  We also show the simulated images of water during irradiation of positive muons with different muon momenta, 73.9-MeV/c, 84.5-MeV/c, and 95.1-MeV/c in lower part of Fig. 3(A), (B) and (C), respectively. We obtained similar to measured images by Monte Carlo simulation.
  The depth profiles measured and simulated in the horizontal direction (X-direction in Fig. 3) are shown in Fig. 4(A) and (B), respectively. The depths of the peaks of the distributions for measured and simulated images are similar; both peaks were located deeper for muons with higher momenta.

1. Optical imaging of water during irradiation of positive muons.
  Measured optical images of water during irradiation of positive muons with different muon momenta, 73.9-MeV/c, 84.5-MeV/c, and 95.1-MeV/c are shown in upper part of Fig. 3(A), (B) and (C), respectively. The muons were irradiated in the water phantom from the left side of the images. We can see elliptical light distributions in all images and these light distributions shifted to the right as the momenta increased. The elliptical light distributions in the images were produced by Cherenkov-light of the positrons in the water from the decay of the positive muons.

**Figure 4:**
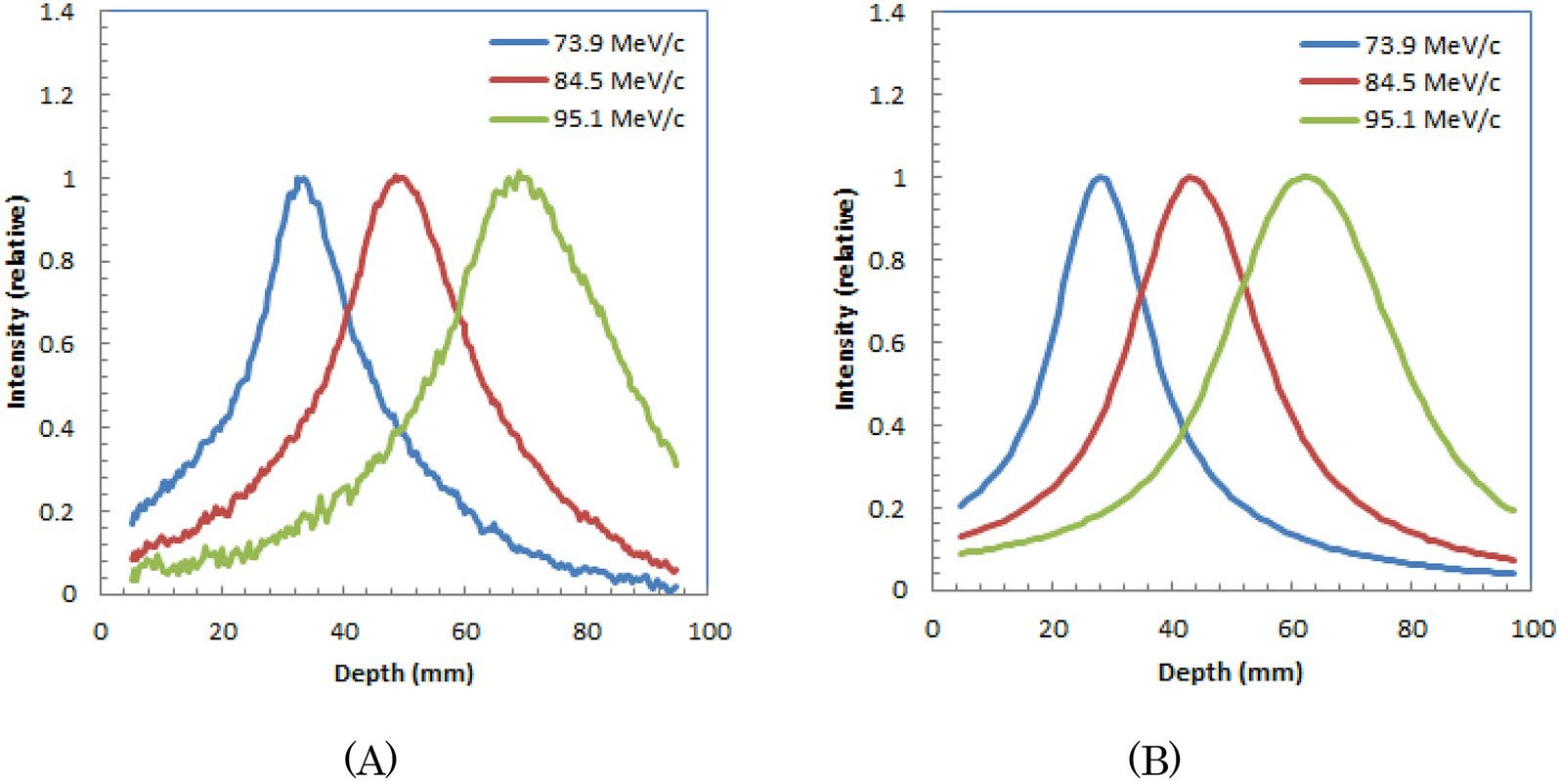
Measured (**A**) and simulated (**B**) depth profiles for optical images of water during 73.9-MeV/c, 84.5-MeV/c, and 95.1-MeV/c irradiation of positive muons.
  Table 1 lists the measured and simulated peak positions from the phantom surface of the water for positive muons with different momenta. We observed that the measured peak positions were larger for muons with higher momenta. The measured values were slightly (4–5 mm) larger than the calculated values.

**Table 1:** Measured and calculated peak positions from the phantom surface for positive muon irradiation to water with different momenta.

Muon momentum (MeV/c)	73.9	84.5	95.1
Measured peak position (mm)	32	48	67
Simulated peak position (mm)	28	43	63
  The lateral profiles measured and simulated for the optical images of water during irradiation of positive muon beams in the vertical direction (Y-direction in Fig. 3) at the maximum intensity position of the images are shown in Fig. 5 (A) and (B), respectively. The widths of the lateral profiles are almost the same, but slightly wider for the muons with higher momenta for both measured and simulated profiles.

**Figure 5:**
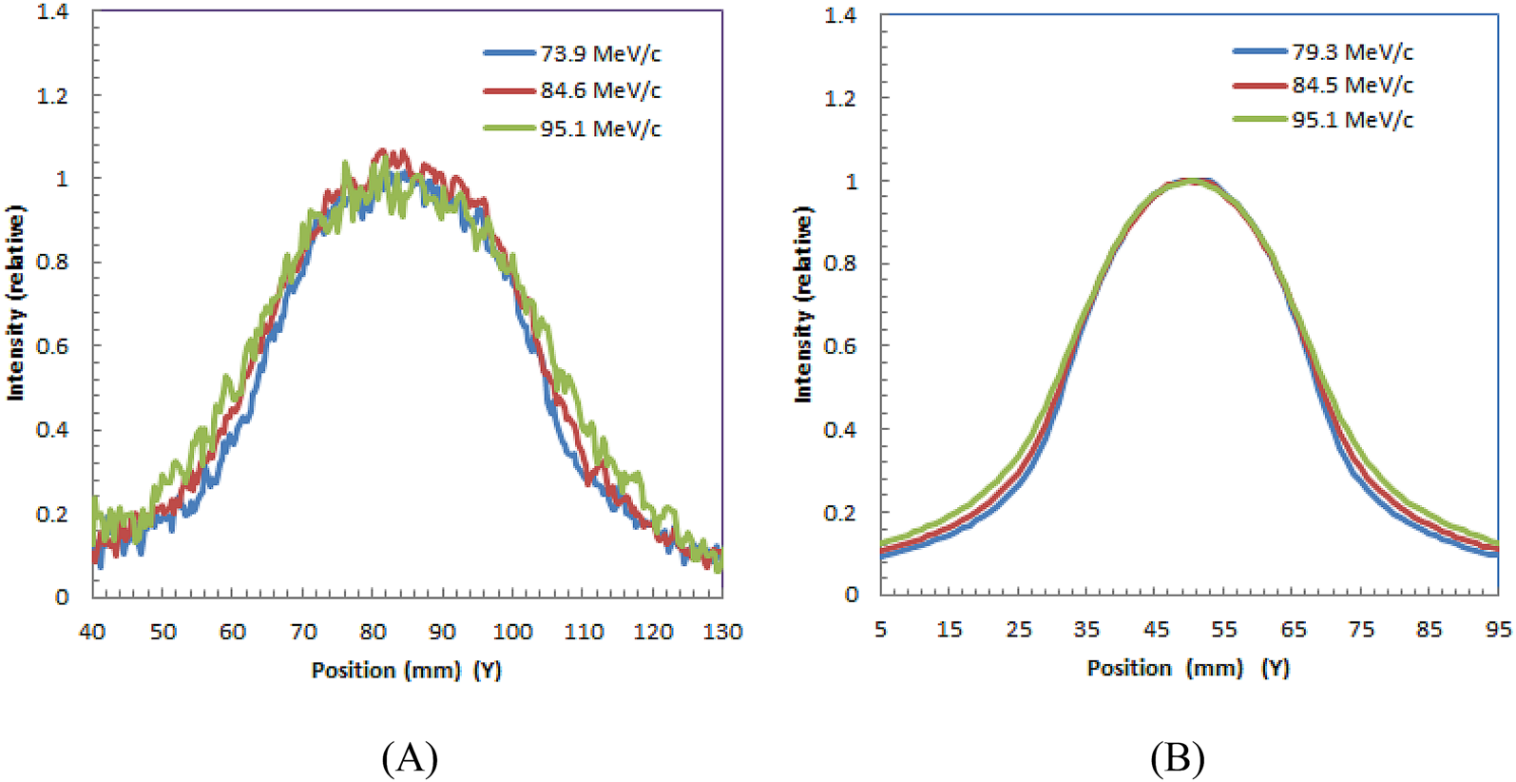
Lateral profiles for optical images of water during 73.9-MeV/c, 84.5-MeV/c and 95.1-MeV/c irradiation of positive muons: measured (A) and simulated profiles (B).
  Table 2 lists the measured and simulated widths for the positive muons in water with different momenta. The widths are slightly larger for muons with higher momenta. The measured widths were slightly (4–8 mm) wider than those of simulated widths.

**Table 2:** Measured and simulated widths for positive muon beams in water with different momenta.

Muon momentum (MeV/c)	73.9	84.5	95.1
Measured width (mm FWHM)	41	45	48
Simulated width (mm FWHM)	37	38	40
2. Optical imaging of plastic scintillator block during irradiation of positive muons.
  Measured optical images of the plastic scintillator block during irradiation of positive muons with different muon momenta, 73.9-MeV/c, 84.5-MeV/c, and 95.1-MeV/c are shown in upper part of Fig. 6 (A), (B) and (C), respectively. The muons were irradiated in the plastic scintillator block from the left side of the images. The light distributions have Bragg peaks at the end of the distributions similar to the dose distributions of particle beams such as protons or carbon ions^8–11^. In all images, the Bragg peak positions shift to the right as the momenta of the muons increase.

**Figure 6:**
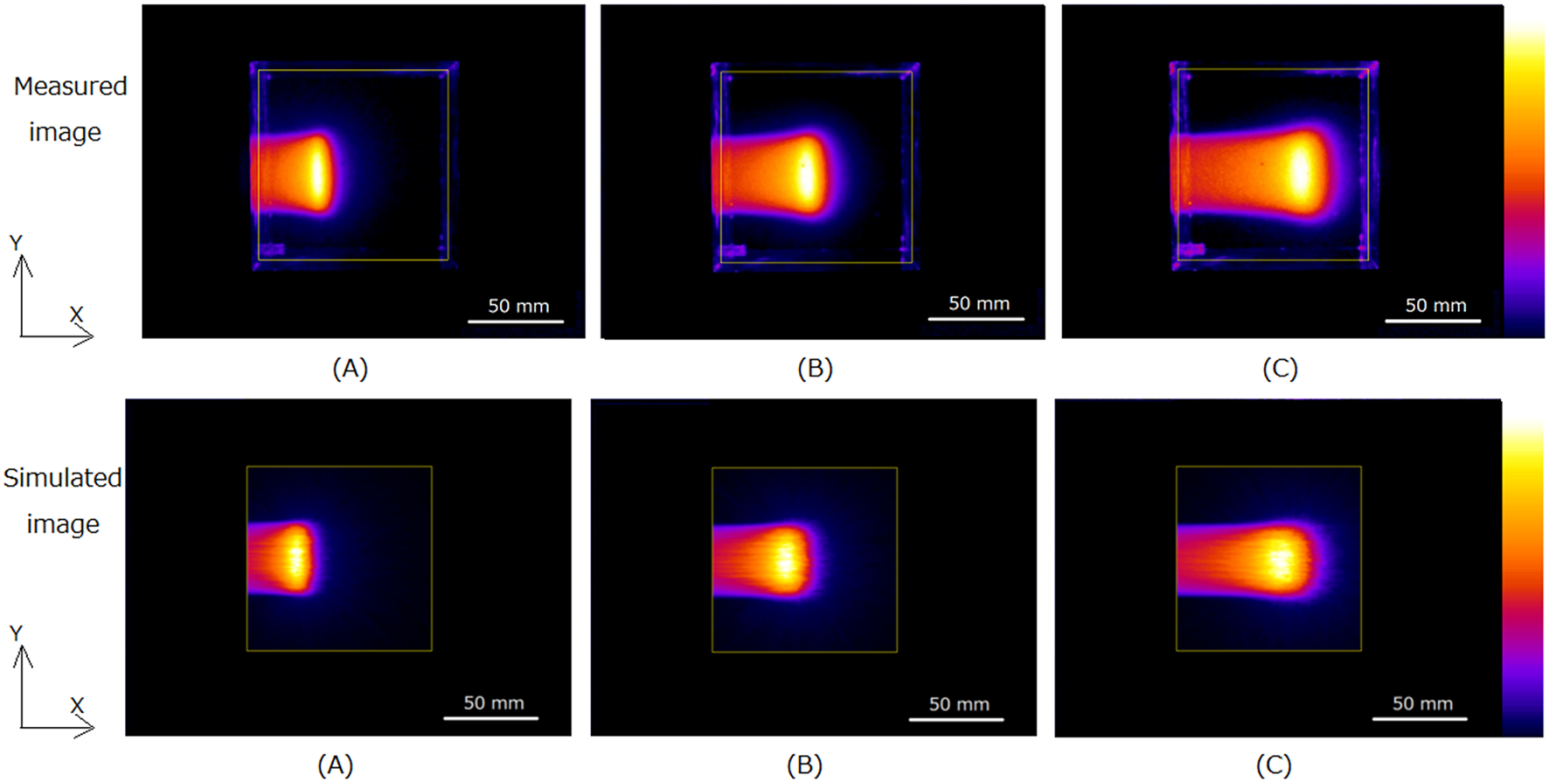
Measured optical images (upper part) of plastic scintillator during irradiation of 73.9-MeV/c (**A**), 84.5-MeV/c (**B**) and 95.1-MeV/c (**C**) positive muons. Background intensities in measured plastic scintillator images were subtracted. Yellow squares in each image show the contours of the plastic scintillator block. Beams were irradiated from left side of phantom. Simulated optical images (lower part) of plastic scintillator during irradiation of 73.9-MeV/c (**A**), 84.5-MeV/c (**B**) and 95.1-MeV/c (**C**) positive muons are also shown.
  We also showed the simulated images of water during irradiation of positive muons with different muon momenta, 73.9-MeV/c, 84.5-MeV/c, and 95.1-MeV/c are shown in lower part of Fig. 6 (A), (B) and (C), respectively. We obtained similar to measured images by Monte Carlo simulation.
  Depth profiles measured and simulated for the horizontal directions (X-direction in Fig. 6) are shown in Fig. 7 (A) and (B), respectively. The depths of the peaks of the distributions for measured and simulated images are similar; both peaks were located deeper for muons with higher momenta.

**Figure 7:**
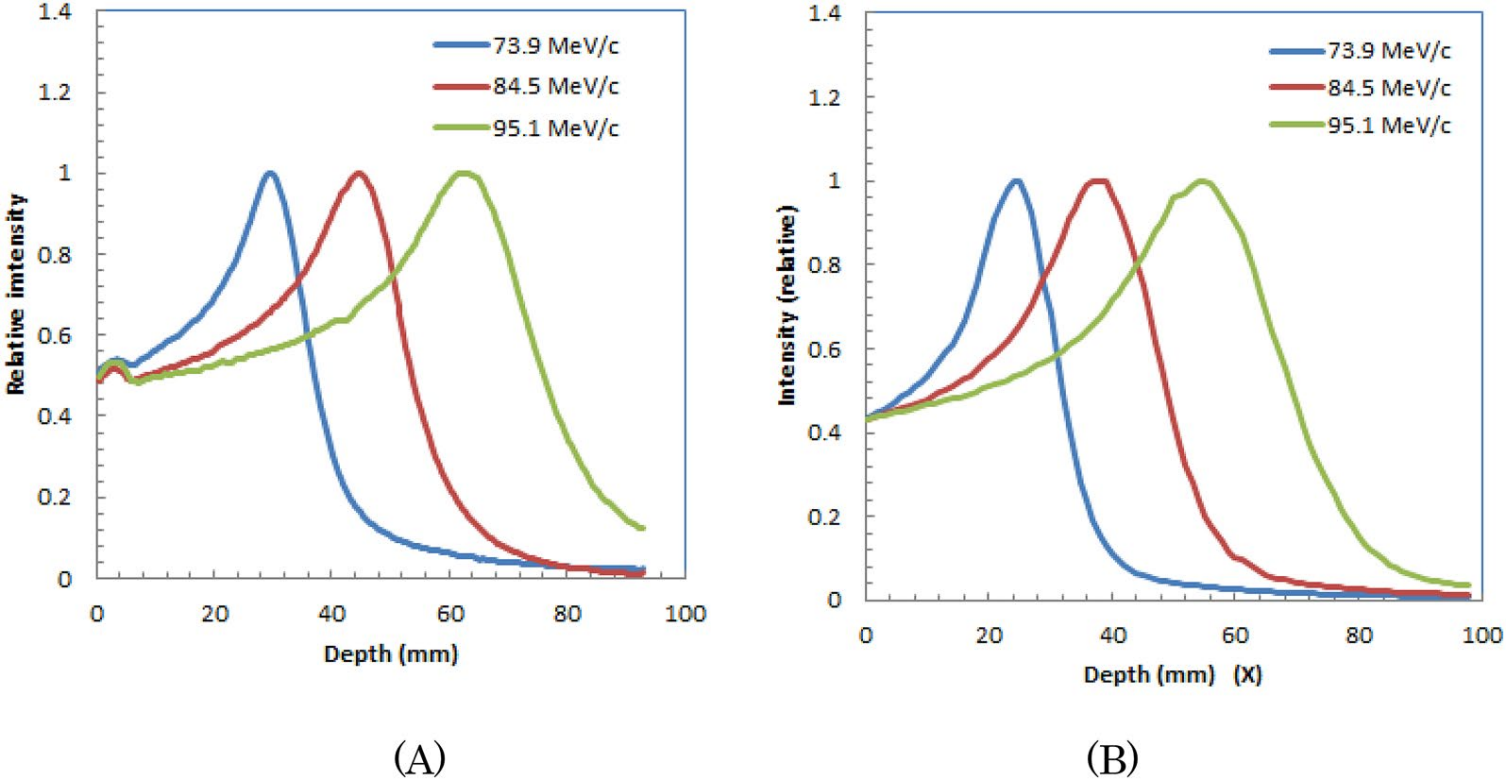
Depth profiles for optical images of plastic scintillator block during 73.9-MeV/c, 84.5-MeV/c, and 95.1-MeV/c irradiation of positive muons: measured (**A**) and simulated profiles (**B**).
  Table 3 lists the measured and calculated peak positions from the block surface for positive muons with different momenta in the plastic scintillator block. The measured peak positions are larger for muons with higher momenta. The measured values were slightly (5–8 mm) larger than the simulated values.

**Table 3:** Measured and simulated peak positions for irradiation of positive muons with different momenta to a plastic scintillator block.

Muon momentum (MeV/c)	73.9	84.5	95.1
Measured peak position (mm)	30	45	62
Simulated peak position (mm)	25	39	54
  In Fig. 8 (A) and (B), we show the lateral profiles (Y-direction in Fig. 6) for measured optical images of the plastic scintillator block during 73.9-MeV/c, 84.5-MeV/c, and 95.1-MeV/c irradiation at 10 mm from the entrance and Bragg peak position, respectively. We observed that the widths were slightly wider at deeper positions for 95.1-MeV/c positive muons. Lateral profiles at 10 mm from entrance and at Bragg peak for simulated optical images are also shown in Fig. 8 (C) and (D), respectively.

**Figure 8:**
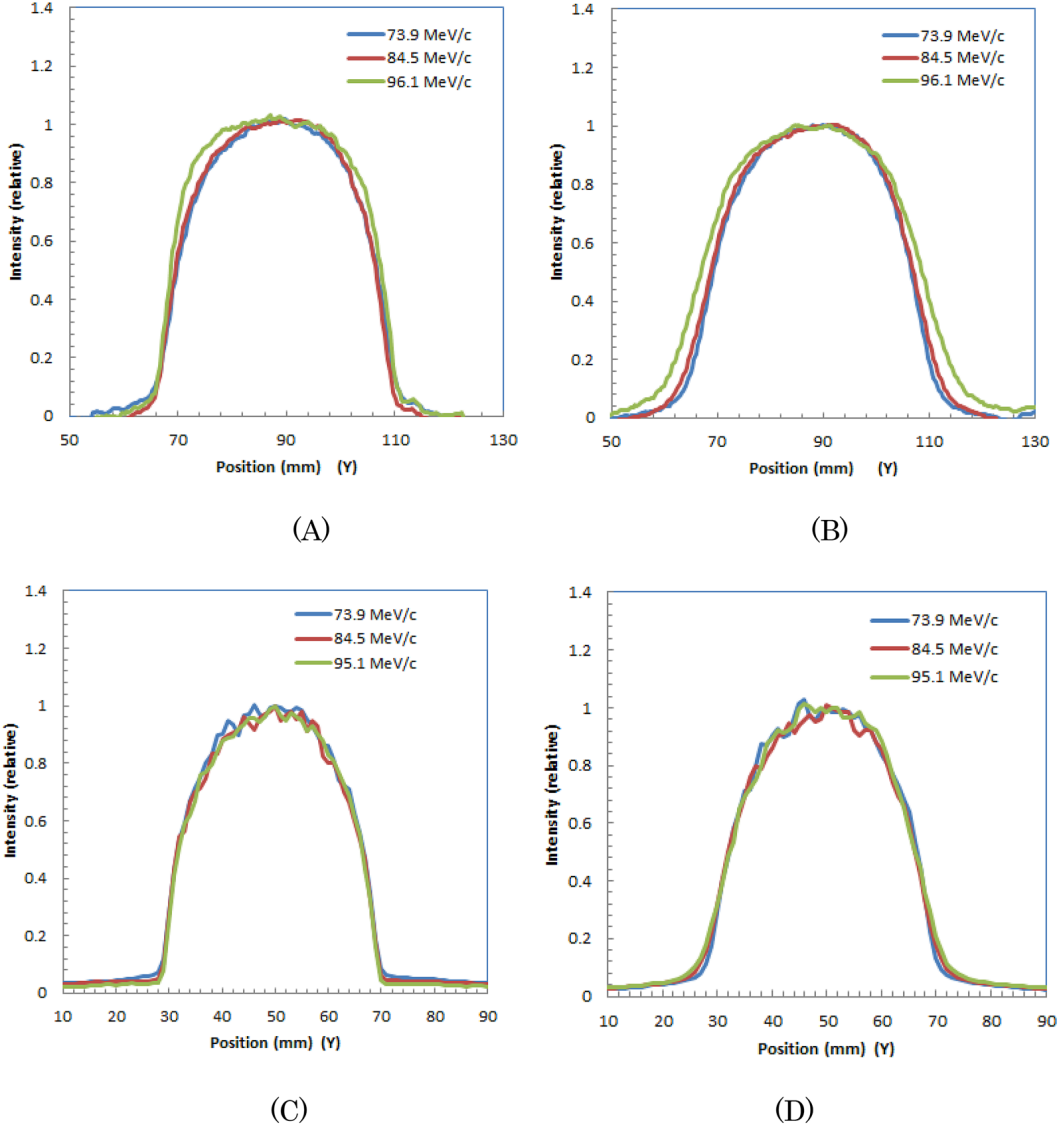
Lateral profiles at 10 mm from entrance (**A**) and at Bragg peak (**B**) for measured optical images of plastic scintillator block during irradiation of 73.9-MeV/c, 84.5-MeV/c, and 95.1-MeV/c positive muons. Lateral profiles at 10 mm from entrance (**C**) and at Bragg peak (**D**) for simulated optical images are also shown.
  Table 4 summarizes the widths at 10 mm from the entrance and Bragg peak positions for positive muons with different momenta in plastic scintillator block images for measured and simulated images. The widths were slightly wider for higher momenta for measured images, and measured widths were slightly wider than simulated ones.

**Table 4:** Measured and simulated widths at 10 mm from entrance and Bragg peak positions for positive muons with different momenta in plastic scintillator block.

Muon momentum (MeV/c)	73.9	84.5	95.1
Measured widths at entrance (mm FWHM)	37	37	39
Simulated widths at entrance (mm FWHM)	35	34	34
Measured widths at Bragg peak (mm FWHM)	37	38	41
Simulated widths at Bragg peak (mm FWHM)	33	33	32
3. Possible applications of the measured images.
  - Beam momentum determination from the images.
    Figure 9 show the relation of the peak depth and the muon momentum evaluated from simulated images of water. This is a proof of concept trial for the possible application. We checked the accuracy of this calculated LUT for the peak depths evaluated from the measured three optical images of water with different momenta shown in Fig. 3. We show the three triangular dots in Fig. 9 for the measured peak depths and muon momenta. They were on the line of water in the graph, indicating the LUT may be used for the beam momenta determination from the peak depths of the measured images.

**Figure 9:**
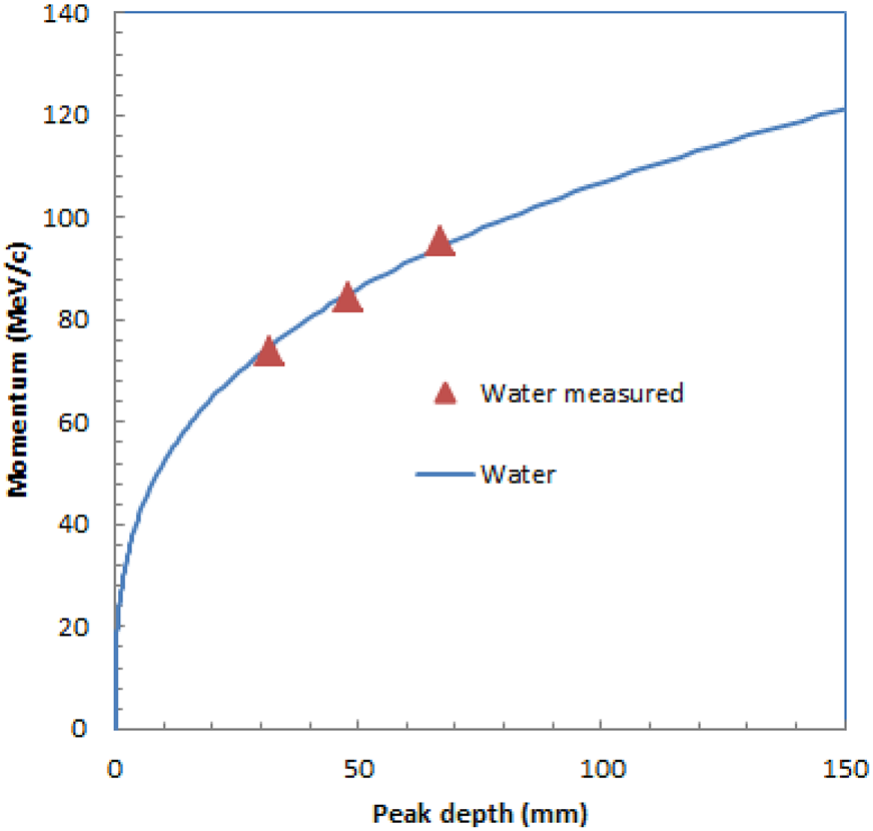
Relation of peak depth of the optical image and muon momentum evaluated from simulated optical images of water during irradiation of positive muons (solid blue lines). Three triangular dots evaluated for measured images of water are also shown.
  - Deviation determination of the momentum of muon beam.
    Figure 10 (A) shows the depth profiles of the simulated images of water with different deviations of momenta for 84.5-MeV/c muons. Figure 10 (B) shows the relation of width and momentum deviation of the muon beam. The width of the measured image is almost on the line as shown in red square dot in Fig. 10 (B). This is also a proof of concept trial for the possible application.

**Figure 10:**
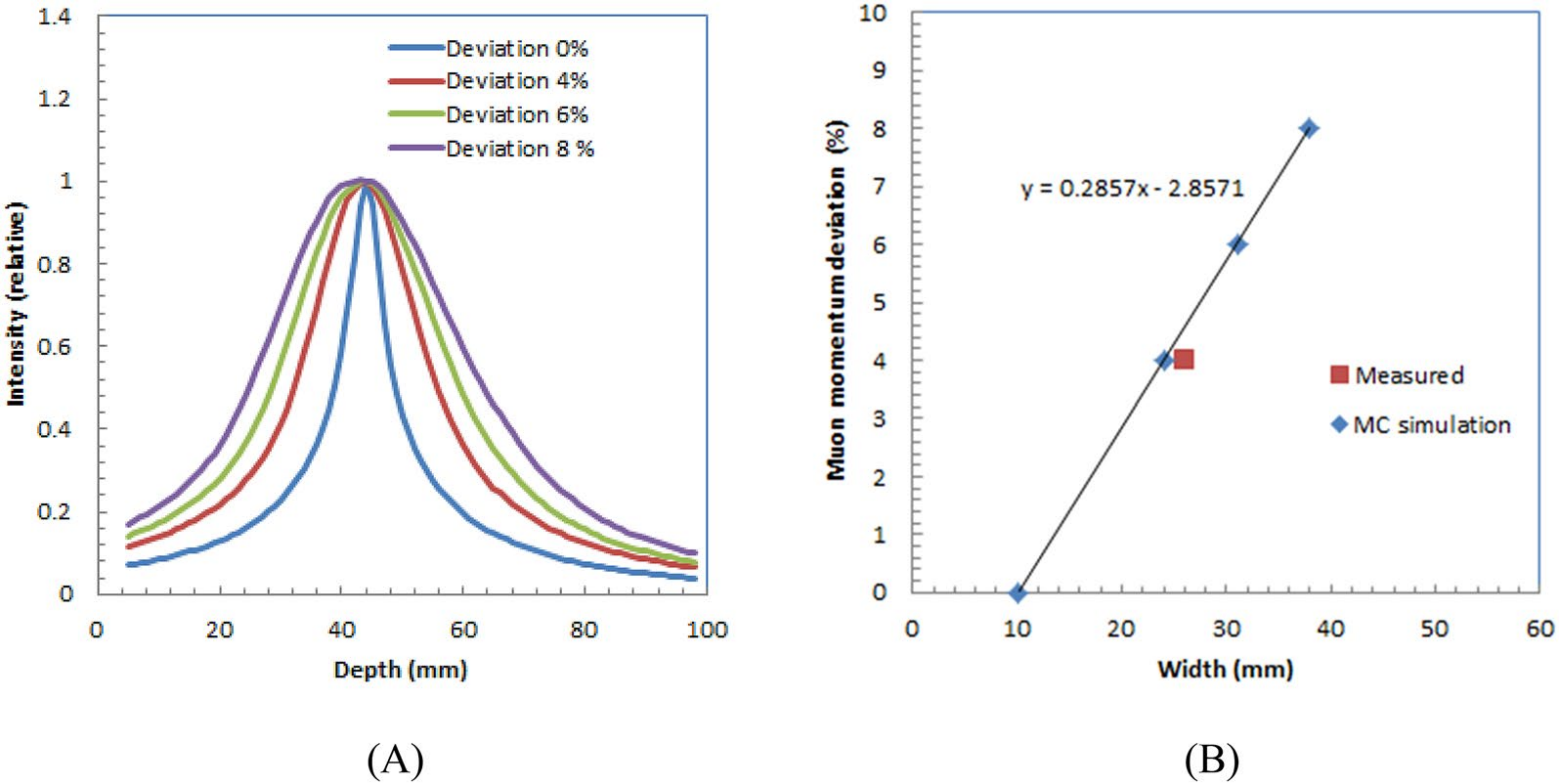
Depth profiles of simulated images of water with different deviations of momenta for 84.5-MeV/c muons (**A**) and relation between width and deviation of muon beam momentum (**B**).
  - Measurement of the asymmetry of the direction of the positron emission from the muon decays.
    Figure 11(A) shows the optical image of water for 84.5-MeV/c measured for 50 min which show a low statistical noise in the image. The depth profile of the image (X-direction) is shown in Fig. 11(B). The distances from the peak shown in A and B of Fig. 11(B) were evaluated to measure the asymmetry of the direction of the positron emission from the muon decays. The calculated B/A was 1.19, that means the distance from the peak in forward direction was 19% longer than that in backward direction. The asymmetry of the direction of the positron emission from the muon decays could be successfully measured. In fact, the 19% larger width in forward direction was within our expected value.

**Figure 11:**
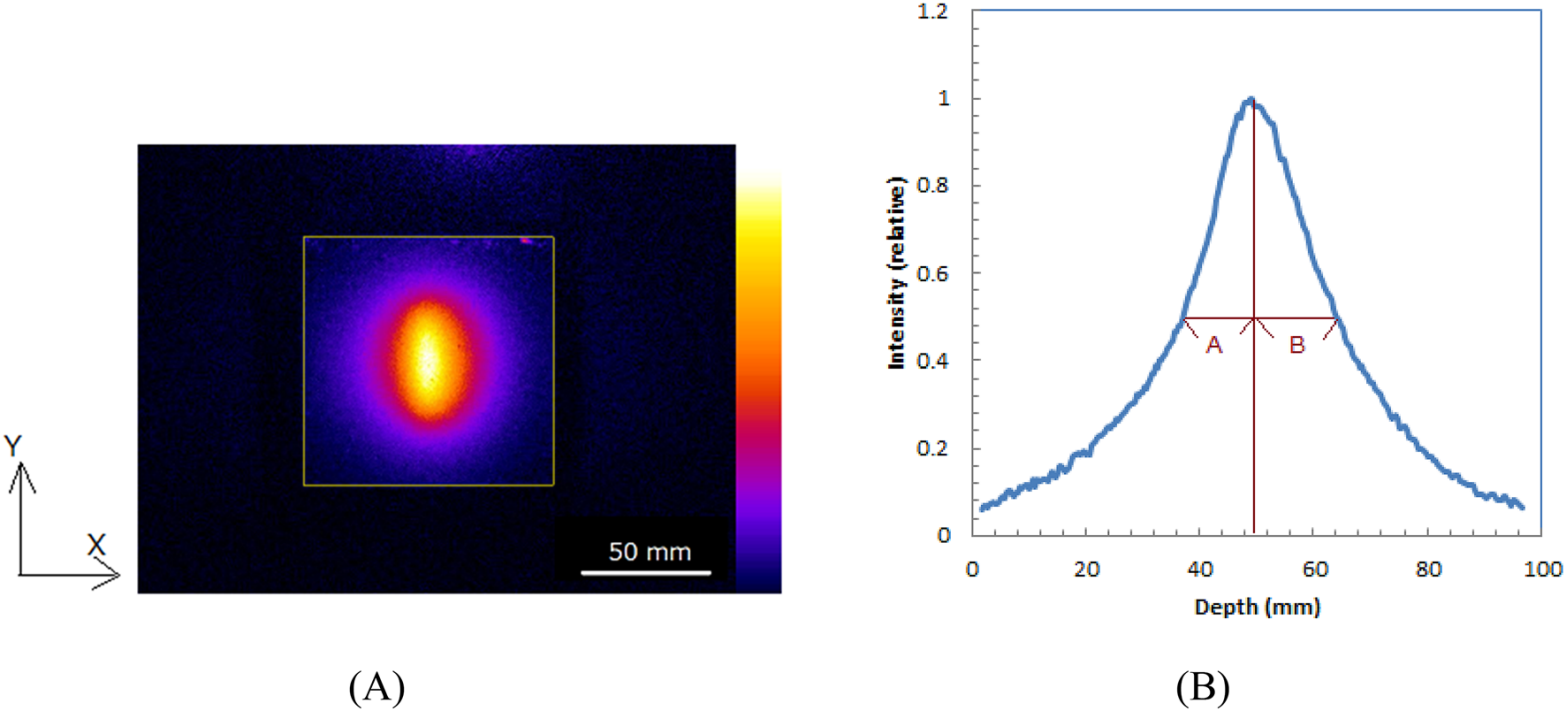
Optical image of water for 84.5-MeV/c measured for 50 min (**A**) and depth profile of image (**B**). Image also shows evaluated distances from peak depth.

## Discussion

We successfully measured the light distributions of water and plastic scintillator block during irradiation of positive muons with different momenta. To the best of our knowledges, these are the world first optical images of muon beams. In water, we obtained elliptical light distributions produced by the Cherenkov-light of positrons from the positive muon decay. We estimated the muon ranges and deviations of the momenta by measuring the peak positions and widths of the images. Estimating the ranges and deviation of momenta in water will be useful for QA of the muon facilities. Estimating the ranges and deviation of momenta in water will also be useful muon radiation therapy in the future because water is the ideal material for QA in radiation therapies.

For the plastic scintillator block, we obtained light distributions having Bragg peaks at the end of the distributions similar to the dose distributions of protons or carbon ions. Since the plastic scintillator block’s light emission is higher than the Cherenkov-light produced in the plastic scintillator block, we obtained similar distributions to dose in the plastic scintillator block. However, since a quenching effect is sometimes observed with a plastic scintillator, which decreases in Bragg peak heights^33,34^, some corrections may be needed to estimate precise dose distributions from the optical images of the plastic scintillator block during muon irradiation. In fact, the relative peak heights of the measured profiles were smaller than those of the simulation. This is also the first result on observation of the quenching effect of muon for plastic scintillator.

The lateral distribution measured in water was wider than those for the plastic scintillator block. This is because the energies of the positrons produced by the decay of the positive muons were high (maximum energy: 52.32 MeV) and the ranges of the positrons in water were longer; thus, the widths of the optical images in water were wider than those of the muon beams. This is consistent with the Monte Carlo simulation results of the Cherenkov-light distribution emitted from the positrons in water^30^.

The measured widths of the muon beams were larger for deeper areas of the plastic scintillator block than those of the shallow areas and the increase in the widths was larger for higher muon energy. This is due to the scatter of the muon beams in the plastic scintillator block. The same phenomenon was more clearly observed in the dose distributions of the muon beams by Monte Carlo simulation for pencil beams^30^. However this is an advantage when the muon beams will be used for mini-beam therapy because the beams need to be wider in Bragg peak area to obtain the flat distributions in the mini-beam therapy^35,36^.

The depth and lateral distributions between measured and simulated images were slightly different both for water and plastic scintillator. The reason was not obvious at this time but we think some improvement of the accuracy of Monte Carlo simulation may be needed for optical imaging of muons. We did not conduct optical corrections such for distortion or parallax errors^37^ in the measured images. These corrections may reduce the differences between measured and simulated images.

The system for optical imaging of water or plastic scintillator block is compact, low cost, and easy to conduct for the imaging. The system could also be used for the momentum determination, the determination of deviation of momentum, and the asymmetry measurements of the directions of the position emission from the decay of muons, although these were the proof of concept trials. Thus the optical imaging will be a promising tool for QA and research tool of the muon beam facilities, as well as future muon beam therapy.

## Conclusions

We measured the light distributions of positive muons in water and a plastic scintillator block. In water, we imaged Cherenkov-light from the positrons around the Bragg peak. The optical images could be used for the muon momentum determination, determination of deviation of momentum, and asymmetry measurements of the directions of the position emission. With the plastic scintillator block, we were able to obtain images similar to dose distributions and estimate beam ranges and widths. For Cherenkov-light images in water, the measured ranges were 4–5 mm larger than the calculated values. From these results, we conclude that optical imaging is promising for QA, research of muon beams, and the future muon radiotherapy.

## References

[1] Glaser AK (2013). Projection imaging of photon beams using Čerenkov-excited fluorescence. Phys. Med. Biol..

[2] Zhang R, Glaser AK, Gladstone DJ, Fox CJ, Pogue BW (2013). Superficial dosimetry imaging based on Čerenkov emission for external beam radiotherapy with megavoltage x-ray beam. Med. Phys..

[3] Demers JL, Davis SC, Zhang R, Gladstone DJ, Pogue BW (2013). Čerenkov excited fluorescence tomography using external beam radiation. Opt Lett..

[4] Glaser AK (2013). Three-dimensional Čerenkov tomography of energy deposition from ionizing radiation beams. Opt. Lett..

[5] Helo Y (2014). Imaging Cerenkov emission as a quality assurance tool in electron radiotherapy. Phys. Med. Biol..

[6] Yamamoto S, Okudaira K, Kawabata F, Nakaya T, Oguchi H (2017). Optical imaging of water during X-ray beam irradiations from linear accelerator. Nuclear Inst. Methods Phys. Res. A.

[7] Yamamoto S, Okudaira K, Kawabata F, Nakaya T, Oguchi H (2018). Imaging of produced light in water during high energy electron beam irradiations from a medical linear accelerator. Radiat. Meas..

[8] Yamamoto S, Toshito T, Okumura S, Komori M (2015). Luminescence imaging of water during proton-beam irradiation for range estimation. Med. Phys..

[9] Yamamoto S (2016). Luminescence imaging of water during carbon-ion irradiation for range estimation. Med. Phys..

[10] Yabe T (2018). Estimation and correction of produced light from prompt gamma photons on luminescence imaging of water for proton therapy dosimetry. Phys. Med. Biol..

[11] Yabe T (2018). Addition of luminescence process in Monte Carlo simulation to precisely estimate the emitted light from water during proton and carbon-ion irradiations. Phys. Med. Biol..

[12] Yamamoto S, Koyama S, Komori M, Toshito T (2016). Luminescence imaging of water during irradiation of X-ray photons lower energy than Čerenkov light threshold. Nuclear Inst. Methods Phys. Res. A.

[13] Yamamoto S (2018). Stability and linearity of luminescence imaging of water during irradiation of proton-beams and X-ray photons lower energy than the Čerenkov light threshold. Nucl. Inst. Methods Phys. Res. A.

[14] Yamamoto S, Komori M, Koyama S, Toshito T (2016). Luminescence imaging of water during alpha particle irradiation. Nucl. Instrum. Methods Phys. Res., Sect. A.

[15] Yamamoto S (2017). Luminescence imaging of water during irradiation of beta particles with energy lower than Čerenkov-light threshold. IEEE Trans. Radiat. Plasma Med. Sci..

[16] Yamamoto S (2018). Source of luminescence of water lower energy than the Čerenkov-light threshold during irradiation of carbon-ion. J. Phys. Commun..

[17] Tanaka HKM (2007). Imaging the conduit size of the dome with cosmic-ray muons: The structure beneath Showa-Shinzan Lava Dome, Japan. Geograph. Res. Lett..

[18] Morishima K (2017). Discovery of a big void in Khufu’s Pyramid by observation of cosmic-ray muons. Nature.

[19] Morishima, K., Naganawa, N., Nakano, T. & Nakamura, M., First demonstration of cosmic ray muon radiography of reactor cores with nuclear emulsion based on an automated high-speed scanning technology. In: Proceedings of the 26th Workshop on Radiation Detectors and Their Uses, 27–36 (2012)

[20] Miyake Y (2012). New muon kicker system for the decay muon beamline at J-PARC. Phys. Proc..

[21] Kadono R, Miyake Y (2012). Reports on progress in physics MUSE, the goddess of muons, and her future. Rep. Prog. Phys..

[22] Ninomiya K (2012). Development of nondestructive and quantitative elemental analysis method using calibration curve between muonic X-ray intensity and elemental composition in bronze. Bull. Chem. Soc. Jpn..

[23] Terada K (2014). A new X-ray fluorescence spectroscopy for extraterrestrial materials using a muon beam. Sci. Rep..

[24] Ninomiya K (2019). Negative muon capture ratios for nitrogen oxide molecules. Radioanal. Nucl. Chem..

[25] Adachi T (2016). Strong electron correlation behind the superconductivity in Ce-free and Ce-Underdoped High-Tc T-Cuprates. J. Phys. Soc. Jpn..

[26] Sugiyama J (2018). Nuclear magnetic field in solids detected with negative-muon spin rotation and relaxation. Phys. Rev. Lett..

[27] Ito TU (2014). Online full two-dimensional imaging of pulsed muon beams at J-PARC MUSE using a gated image intensifier. Nucl. Instrum. Methods A.

[28] Daniel H (1969). The muon as a tool for scanning the interior of the human body. Nuclear-Medizin.

[29] Mokhov, N. V. & Van Ginneken, A., Muons versus hadrons for radiotherapy. In: Proceedings of the 1999 Particle Accelerator Conference, 2525–2527 (1999)

[30] Hirano Y, Ninomiya K, Yamamoto S (2019). Estimation of dose and light distributions in water during irradiation of muon beams. Phys. Scr..

[31] Nagamine K (1982). Long-lived muonium in water revealed by pulsed muons. J. Chem. Phys. Lett..

[32] Bayes R (2011). Experimental constraints on left-right symmetric models from muon decay. Phys. Rev. Lett..

[33] Robertson D, Mirkovic D, Sahoo N, Beddar S (2013). Quenching correction for volumetric scintillation dosimetry of proton beams. Phys. Med. Biol..

[34] Beddar AS, Mackie TR, Attix FH (2009). Exploration of the potential of liquid scintillators for real-time 3D dosimetry of intensity modulated proton beams. Med. Phys..

[35] Prezado Y, Fois GR (2013). Proton-minibeam radiation therapy: A proof of concept. Med. Phys..

[36] Dilmanian FA, Eley JG, Krishnan S (2015). Minibeam therapy with protons and light ions: Physical feasibility and potential to reduce radiation side effects and to facilitate hypofractionation. Int. J. Radiat. Oncol. Biol. Phys..

[37] Yabe T, Komori M, Horita R, Toshito T, Yamamoto S (2018). Estimation of the optical errors on the luminescence imaging of water for proton beam. Nuclear Inst. Methods Phys. Res. A.

